# *Pseudomonas aeruginosa* biofilm is a potent inducer of phagocyte hyperinflammation

**DOI:** 10.1007/s00011-019-01227-x

**Published:** 2019-03-18

**Authors:** Marta Ciszek-Lenda, Magdalena Strus, Maria Walczewska, Grzegorz Majka, Agnieszka Machul-Żwirbla, Diana Mikołajczyk, Sabina Górska, Andrzej Gamian, Benjamin Chain, Janusz Marcinkiewicz

**Affiliations:** 1 Chair of Immunology, Jagiellonian University Medical College, Kraków, Poland; 2 Chair of Microbiology, Jagiellonian University Medical College, Kraków, Poland; 3 Ludwik Hirszfeld Institute of Immunology and Experimental Therapy, Polish Academy of Sciences, Wrocław, Poland; 4 Division of Infection and Immunity, UCL, London, UK

**Keywords:** Biofilm, Hyperinflammation, Neutrophils, *P. aeruginosa*, LPS, DNA

## Abstract

**Objective:**

*Pseudomonas aeruginosa* effectively facilitate resistance to phagocyte killing by biofilm formation. However, the cross talk between biofilm components and phagocytes is still unclear. We hypothesize that a biofilm provides a concentrated extracellular source of LPS, DNA and exopolysaccharides (EPS), which polarize neighbouring phagocytes into an adverse hyperinflammatory state of activation.

**Methods:**

We measured the release of a panel of mediators produced in vitro by murine neutrophils and macrophages exposed to various biofilm components of *P. aeruginosa* cultures.

**Results:**

We found that conditioned media from a high biofilm-producing strain of *P. aeruginosa*, PAR5, accumulated high concentrations of extracellular bacterial LPS, DNA and EPS by 72 h. These conditioned media induced phagocytes to release a hyperinflammatory pattern of mediators, with enhanced levels of TNF-α, IL-6, IL12p40, PGE_2_ and NO. Moreover, the phagocytes also upregulated COX-2 and iNOS with no influence on the expression of arginase-1.

**Conclusions:**

Phagocytes exposed to biofilm microenvironment, called by us biofilm-associated neutrophils/macrophages (BANs/BAMs), display secretory properties similar to that of N1/M1-type phagocytes. These results suggest that in vivo high concentrations of LPS and DNA, trapped in biofilm by EPS, might convert infiltrating phagocytes into cells responsible for tissue injury without direct contact with bacteria and phagocytosis.

## Introduction

*Pseudomonas aeruginosa* is an opportunistic human pathogen that preferentially attacks immunocompromised patients and causes diverse chronic infections, such as severe nosocomial pneumonia and non-healing wounds [1–3]. Chronic lung infection with *P. aeruginosa* is a highly morbid complication that is particularly found in patients with cystic fibrosis (CF) [4]. *P. aeruginosa* has strong pathogenicity and resistance to antibiotic therapy, largely due to its inherent biofilm-forming capacity [5]. Bacterial infections associated with biofilm formation are characterized by severe and progressive chronic inflammation, massive neutrophil infiltration but paradoxical resistance to immune attack [6, 7]. Two explanations for the increased resistance of biofilm-forming bacteria have been put forth: (1) bacterial cells may be hidden in the biofilm matrix, such that their contact with antibacterial agents or immune cells is reduced; or (2) infiltrating phagocytes may be less effective in killing biofilm-hidden pathogens [8].

Mechanisms of tissue injury and chronic inflammation during chronic *P. aeruginosa* infections may reflect direct cytotoxic effects of *P. aeruginosa* toxins (e.g., rhamnolipids) and indirect effects of immunopathogenic mediators (e.g., NO, ROS, TNF-α) released from infiltrating phagocytes [9]. Biofilm formation is characterized by pronounced metabolic changes of sessile bacteria and secretion of strain-specific extracellular polymeric substances, including exopolysaccharides (EPSs), DNA, LPS and proteins [10–12]. These biofilm matrix components may, therefore, provide a concentrated extracellular source of PAMPS, which activate and polarize biofilm-infiltrating neutrophils and macrophages into more aggressive proinflammatory subtypes [13, 14]. Biofilm may, therefore, both protect bacteria from killing, yet activate hyperinflammation. It has been well documented that hyperinflammatory response, the effect of activation of neutrophils and macrophages with limited access to targets, does favor chronic inflammation without effective bacterial clearance [15–18]. Such reaction was also named as “*frustrated phagocytosis*” [19, 20].

We hypothesize that neutrophils and macrophages, upon contact with active biofilm matrix components such as found in a biofilm environment, polarize into biofilm-associated neutrophils (BANs) and biofilm-associated macrophages (BAMs). Based on previous studies [15, 21, 22], we suggest that these phagocytes will be of hyperinflammatory phenotype, sometimes referred to as N1/M1 phenotypes. We explore this hypothesis by examining the inflammatory phenotype of macrophages and neutrophils exposed to *P. aeruginosa* extracellular products produced early (8 h) in culture, when biofilm production is just initiated, and *P. aeruginosa* extracellular products produced late (72 h), when massive biofilm production takes place.

## Materials and methods

### Characterization of *P. aeruginosa* strain PAR5

All experiments in this study were performed on mucoid *P. aeruginosa* strain coded as PAR5, which was selected from a larger collection out of 20 *P. aeruginosa* strains isolated from patients with chronic non-healing diabetic foot infections. PAR5 has the ability to form the largest amount of biofilm mass compared to the same population of other strains from the collection [23, 24].

#### Growth conditions and measurement of biofilm formation by PAR5

The initial culture of PAR5 was propagated in 10 ml of tryptic soy broth (TSB, Difco) at 37 °C for 24 h under aerobic conditions. After cultivation, bacteria were centrifuged for 10 min at 500*g* and washed with 10 ml of phosphate buffered saline (PBS, pH 7.4, Sigma-Aldrich). Bacterial count, confirmed by decimal dilutions, reached 1 × 10^8^ CFU/ml.

**Fig. 1:**
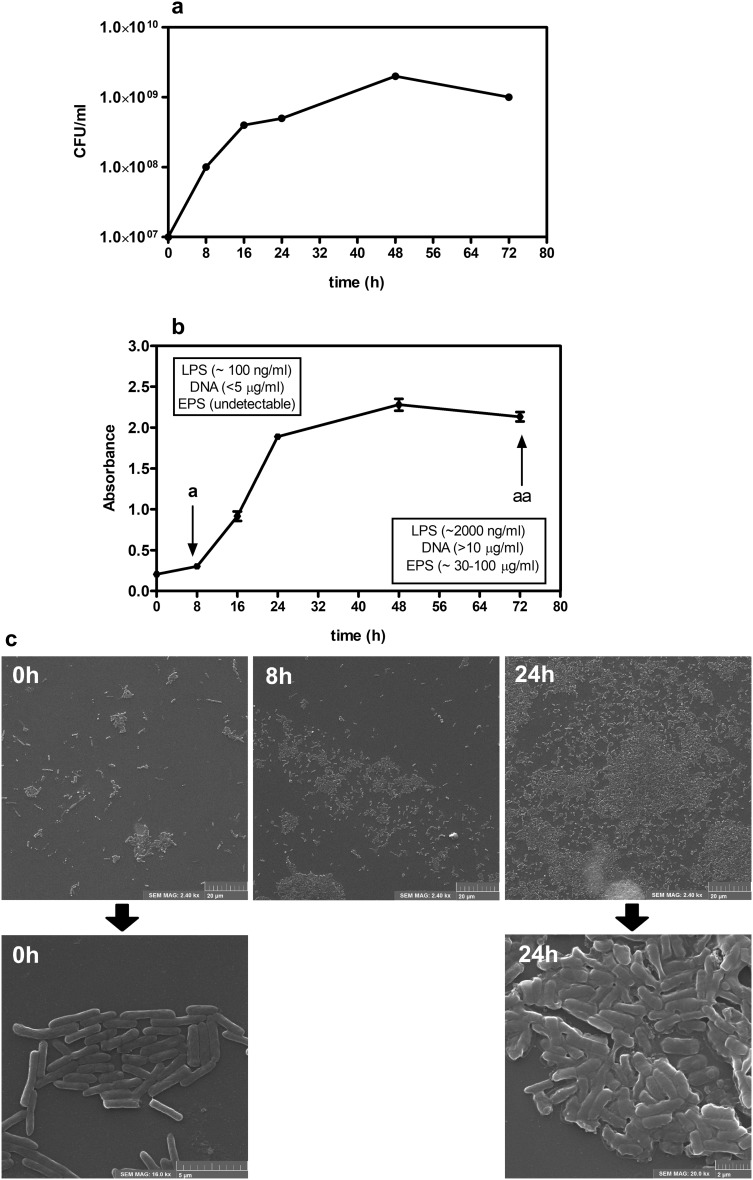
The representative record of kinetics of biofilm formation by PAR5. **a** Density of PAR5 population at the indicated points of time is expressed in CFU of sessile cells per ml. The culture started with the bacterial suspension at a density of 10^7^ CFU/ml. **b** Building of biofilm matrix. Congo red staining was used for measuring biofilm formation (EPS accumulation). Plot depicts mean values ± SD of absorbance (*λ* = 492 nm). The mean was calculated on the basis of three independent experiments (*n* = 3). ^a^8-h PAR5 planktonic bacteria cell attachment and the beginning of biofilm formation ^aa^72 h—mature biofilm. At these time points of the culture, the concentration of DNA, LPS and EPS in bacterial conditioned media is shown. **c** SEM of: 0-h PAR (free-floating planktonic bacteria); 8-h PAR5 (cluster of planktonic bacteria, the beginning of biofilm formation); 24-h PAR5 (conglomerate cluster of bacteria—early biofilm)

Biofilm quantity (total mass of bacterial polysaccharides) was measured in sterile plastic 96-well plates with adherent surface (Greiner Bio-One) using Congo red dye according to a modified procedure described by Reuter et al. [25]. Briefly, 20 µl of fresh culture of the bacterial suspensions, prepared as described above, was added to each well followed with 180 µl of sterile TSB. Final concentration of the bacteria was 1 × 10^7^CFU/ml. The plates were centrifuged for 10 min at 500*g* to sediment bacteria at the bottom of each well. Bacteria were then incubated for 72 h (37 °C, aerobic conditions). At different time points of the culture (0, 8, 16, 24, 48 and 72 h), the plates were centrifuged, the culture medium was gently removed from wells and, immediately, 200 µl of 0.1% Congo red solution was added. The plates were left for 30 min at room temperature (RT) and washed twice with buffered saline to remove unbound dye. Absorbance was measured at *λ* = 492 nm wavelength using a spectrophotometer (Awareness Technology Inc.). All measurements were performed in triplicates and mean values ± SD were given. The representative record of kinetics of bacteria growth and biofilm formation by PAR5 is shown in Fig. 1a, b. Moreover, SEM images of PAR5 cultures (0, 8, and 24 h) were prepared (Fig. 1c). Bacterial morphology was examined under a scanning electron microscope (TESCAN). Coverslips were placed in a 12-well plate and treated with poly-l-lysine to enhance further bacterial cell adhesion. Glass slides were immersed in bacterial suspensions for 0, 8, and 24 h. Then, 1 ml of fixing buffer (sodium cacodylate in 2.5% glutaraldehyde and 0.1 M sucrose) was added and the slides were incubated for 1.5 h at 37 °C. A dehydration procedure was applied in a gradient of methanol. Dried samples were sputtered with a 20-nm gold layer (Quorum, Q150R S) to facilitate SEM visualization [26].

### PAR5 culture and preparation of bacterial-conditioned media

1 ml of the bacterial suspension, prepared as described above, was added to sterile tubes, each containing 9 ml of fresh TSB broth, and cultured for either 8 h (planktonic stage of PAR5 cultures) or 72 h (mature biofilm stage of PAR5 cultures). The bacteria were grown under aerobic conditions without shaking at 37 °C.

#### PAR5-8 h

After 8 h, the number of PAR5 cells present in the culture was estimated by making an appropriate decimal dilution of the bacteria in PBS and counting the bacterial colonies plated on McConkey agar (Oxoid) (Fig. 1a). The remaining volume of the PAR5 culture was centrifuged for 10 min at 500*g*. Supernatant was transferred to a fresh tube, filtered with a 0.22-µm Millipore membrane, and stored at 4 °C until needed. This solution was labeled as BCM-8 h (planktonic bacterial-conditioned medium). The pellet of PAR5-8 h bacterial cells was washed with 10 ml PBS (pH 7.4) and stored at 4 °C.

#### PAR5-72 h

The above steps were repeated after 72 h to collect PAR5-72 h bacterial cells and BCM-72 h (mature biofilm-conditioned medium) for further studies.

### Killing *P. aeruginosa* PAR5 bacterial cells

PAR5 pellets originating from the 8- and 72-h cultures were treated thrice with high temperature (121 °C) at 0.3 bars in the ASVE-ELMI ESS-207 SMS steam sterilizer. The follow-up culture was verified to be sterile.

### Measurement of bacterial DNA in bacterial condition media

Extracellular bacterial DNA was isolated and purified from bacterial-conditioned media, according to the manufacturer’s protocol (Genomic Mini, A&A Biotechnology, Poland). Concentration of purified, specific-density DNA was determined with a spectrophotometer (DS-11; DeNovix Co.) at a wavelength of 260 nm.

### Measurement of LPS concentration in bacterial-conditioned media

Level of LPS in tested samples was estimated by use of QCL-1000 Chromogenic LAL test (Lonza) according to the manufacturer’s protocol. QCL-1000 is an assay for the detection of Gram-negative bacterial endotoxin.

### DNA extraction

The bacterial cells from 72 h grown culture were harvested (8000 rpm, 10 °C, 15 min). The pellet was washed twice by PBS and again harvested. 0.25 ml of 10 mM Tris–HCl (pH 8) and 2.5 mg/ml of lysozyme were added and incubated at 37 °C for 2 h. Then, 0.5 ml of lysis buffer (50 mM Tris, 100 mM EDTA, 1% SDS, pH 8) and 1 mg/ml of proteinase-K were added and incubated at 50 °C for 2 h in a water bath. The digestion with proteinase-K was followed by addition of 0.5 ml of phenol:chloroform (1:1). The samples were mixed gently for 3 min and harvested (14,000 rpm, 4 °C, 15 min). The upper layer was transferred in fresh tube and extracted with chloroform:isoamyl alcohol (24:1) by the centrifugation at 14,000 rpm on 4 °C for 15 min. This step was repeated. The supernatant was precipitated with twice volume of ethanol and left till the precipitation was settled down. DNA was collected by centrifuging at 14,000 rpm, on 4 °C for 15 min and dried. The pellet was suspended in 10 mM Tris–HCl (pH 8) and 1 mM EDTA (pH 8) buffer and incubated at 45 °C in a water bath for 3 h. The quality of DNA was checked using DS-11 spectrophotometer (DeNovix).

### LPS isolation

LPS was isolated using hot phenol/water method and purified as described by Westphal et al. [27]. Quantity of LPS was measured after the sample lyophilisation.

### EPS isolation

EPS was isolated as described previously [28]. The obtained material was purified by DNAse, RNAse and protease. Total saccharide concentration was measured by phenol sulfuric acid method according to Dubois’s method [29].

### Fractionation of bacterial-conditioned media

BCM-72 h in volumes of 2 ml was placed in Sartorius fractionation columns (Vivacon 2) with molecular weight (MW) cut-off of 10 kDa and centrifuged for 30 min at 3500*g* at 4 °C. Then, the lower fraction which was < 10 kDa MW was collected. Then, the columns were turn over and centrifuged once more for 3 min at the same conditions to restore > 10 kDa MW fraction of BCM-72 h.

### Mice

Inbred CBA mice (8–12 weeks of age, 18–22 g) were maintained at the Animal Breeding Unit of the Department of Immunology of Jagiellonian University Medical College. All mice were held in standard caging conditions with water and standard diet ad libitum. This study was carried out in strict accordance with recommendations from the Guide for the Care and Use of Laboratory Animals of the Ministry of Science and Information of Poland. The protocol was approved by the I Local Committee on the Ethics of Animal Experiments of Jagiellonian University. All surgeries were performed under isoflurane (Abbott Laboratories) anesthesia. Every effort was made to minimize animal suffering. Mice were used as donors for peritoneal exudate cells.

### Cell isolation

Peritoneal mouse exudate cells (PEC) were induced by an intraperitoneal injection of 1.5 ml of 3% thioglycollate (Difco Laboratories). Mice were euthanized by overdosing with isoflurane vapors (Abbott Laboratories), followed by cervical dislocation. PEC were collected by washing out the peritoneal cavity with 5 ml of PBS (Lonza) containing 5 U heparin/ml (Polfa). Then, PEC were centrifuged, and red blood cells were lysed. Osmolarity was restored by addition of PBS. To obtain neutrophils, PEC were collected after 18 h or for macrophages 96 h after thioglycollate injections. For neutrophil collections, we have used 18-h thioglycollate-induced peritoneal exudate cells. The cells were seeded to plastic plates and after 1 h, we collected non-adherent cells (neutrophils) for further experiments. For peritoneal macrophage isolation, we have used the method described previously [30]. At least three mice were used as donors of PEC for each experiment.

### Cell culture and treatment

Neutrophils and macrophages were cultured in 24-well flat-bottom cell culture plates at 5 × 10^5^/well in IMDM medium (Lonza) supplemented with 5% fetal bovine serum (FBS; Lonza), 2 mM stable l-glutamine (Cytogen), and 50 mg/ml gentamicin (KRKA) at 37 °C in an atmosphere of 5% CO_2_. To determine the influence of the biofilm forms of *P. aeruginosa* on innate immune cell activity, neutrophils and macrophages were stimulated with heat-killed whole bacterial cells (PAR5-72 h 20:1 and 100:1 bacteria per cell) or with 72-h conditioned media from bacterial cultures (BCM-72 h at 5–20% total volume), if not otherwise stated. The effect was compared with that of bacterial cells (PAR5-8 h) and 8-h conditioned media (BCM-8 h). As a reference stimulus for N1/M1 neutrophils/macrophages, we used 100 ng/ml LPS from *Escherichia coli* strain 0111:B4 (LPS, Sigma-Aldrich). After 24 h of stimulation, culture supernatants were collected and frozen at − 80 °C until use. Cells were used in a western blot analysis. In some experiments, neutrophils were cultured with BCM-72 h treated with inhibitors of DNA (BCM were incubated in the presence of 2.5 µl of 1500 Kunitz DNAse I, Qiagen; chloroquine, Sigma-Aldrich—2.5 µg/ml) or/and with the inhibitor of LPS (polymyxin B, Sigma-Aldrich, 100 µg/ml) or preincubated with EPS (30 µg/ml) for 1 h and then re-stimulated with LPS (100 ng/ml) or DNA (3 µg/ml).

### Cell viability

Cell viability was monitored by mitochondrial-dependent reduction of MTT (Promega) to formazan and by means of LDH activity (lactate dehydrogenase) using LDH assay kit (Pierce) according to manufacturer’s instruction. The viability of phagocytes was controlled in all experimental systems to avoid cytotoxic effect of the tested agents. The lowest viability of neutrophils was approximately 78% (24 h after exposition to DNA), in other groups, the viability of neutrophils was ~ 80% and macrophages ~ 90%.

### Cytokine determination

Cytokine levels in cell culture supernatants were measured by sandwich ELISA. Microtiter plates (Costar EIA/RIA plates, Corning Inc.) were coated with a cytokine-specific antibody. Expression levels of IL-6, IL-10, and IL-12p40 were measured according to the manufacturer’s instructions (OptEIA Sets, BD Biosciences). TNF-α level was measured according to the manufacturer’s instructions (ELISA Ready-Set-Go, eBioscience). In all cases, 10% FBS in PBS was used as a blocking solution. TMB substrate solution (BioLegend) was used to develop a colorimetric reaction, which was stopped with 2 M sulfuric acid. Optical density was measured at 450 (570) nm using a microtiter plate reader (PowerWaveX, Bio-Tek Instruments).

### Nitric oxide (NO) determination

NO levels in culture supernatants of peritoneal thioglycollate-induced cells (neutrophils, macrophages) were quantified by the accumulation of nitrite as a stable end product, according to a modified Griess method [31]. Cell culture supernatant (100 µl) was mixed with 14 mM 4,4′-diamino-diphenylsulphone (Dapsone, Sigma-Aldrich) in 2 M HCl (50 µl) and 0.1% *N*-1-naphtylenediamine dihydrochloride (50 µl) in deionized water. Absorbance of the tested culture supernatants at 550 nm was compared with a sodium nitrate standard (NaNO_2_) curve.

### Prostaglandin E_2_ (PGE_2_) immunoassay

PGE_2_ concentration in cell supernatants was determined by a PGE_2_ high-sensitivity ELISA kit (Enzo Life Sciences), according to the manufacturer’s protocol.

### Western blot analysis

24 h after in vitro stimulation of macrophages or neutrophils, expression levels of COX-2, iNOS, and arginase-1 (Arg-1) proteins in cell cytosol were determined by western blot analysis. After incubation, cells were lysed in lysis buffer (1% Triton X-100, 0.1% SDS in PBS) containing protease inhibitor cocktail (Sigma-Aldrich). Protein concentrations in lysates were determined using a bicinchoninic acid protein assay kit (Sigma-Aldrich). Samples containing equal amounts of total protein were mixed with gel loading buffer (0.125 M Tris, 4% SDS, 20% glycerol, 0.2 M dithiothreitol, 0.02% bromophenol blue) in a 2:1 ratio (v/v) and boiled for 4 min. Samples of 20 µg of total protein per lane were separated on 10% SDS-polyacrylamide gels (Mighty Small II, Amersham Biosciences) using the Laemmli buffer system. Proteins were transferred to nitrocellulose membranes (Bio-Rad). Non-specific binding sites were blocked overnight at 4 °C with 3% non-fat dried milk. Membranes were incubated for 2 h at RT with rabbit polyclonal antibodies to COX-2 (cat. no. 160106, 1:1000, Cayman), Arg-1 (clone H-52, 1:600, Santa Cruz Biotechnology), or iNOS (ADI-KAS-NO001, 1:2000, Enzo Life Sciences). Bands were detected with alkaline phosphatase-conjugated secondary goat antibody to the rabbit IgG whole molecule (A9919, 1 h at RT, 1:3000, Sigma-Aldrich) and developed with BCIP/NBT alkaline phosphatase substrate (Sigma-Aldrich). Membranes were re-probed with monoclonal mouse anti β-actin antibody (clone AC-15, 1 h at RT, 1:3000, Sigma-Aldrich). Pre-stained SDS-PAGE standards (low and high range; Bio-Rad) were used for molecular weight (MW) determinations. Protein bands were scanned and analyzed with the Scion Image freeware (Scion Corp.). Data were normalized to the constitutive expression level of β-actin protein.

### Data analysis

Statistical significance of differences between groups was analyzed using one-way ANOVA, followed, if significant, by a Dunnett’s test for post hoc comparison. In some experiments, Student’s *t* test was used. Results are expressed as mean ± SEM values. A *P* value < 0.05 was considered statistically significant. Analysis was performed using Graphpad Prism v. 5.01 (GraphPad Software, Inc.). The exact statistical analysis is named in the relevant figure’s capitation.

## Results

### Cytokine production by cells exposed to *P. aeruginosa* PAR5

*Pseudomonas aeruginosa* switches from a planktonic to a biofilm-producing stage during prolonged culture in vitro. We exposed neutrophils and macrophages to killed bacteria cells (PAR5) or bacterial-conditioned media isolated from bacterial cultures after 8 h beginning of biofilm formation—planktonic stage of bacteria growth—see SEM images (Fig. 1) or 72 h (biofilm matrix formation—mature biofilm).

**Fig. 2:**
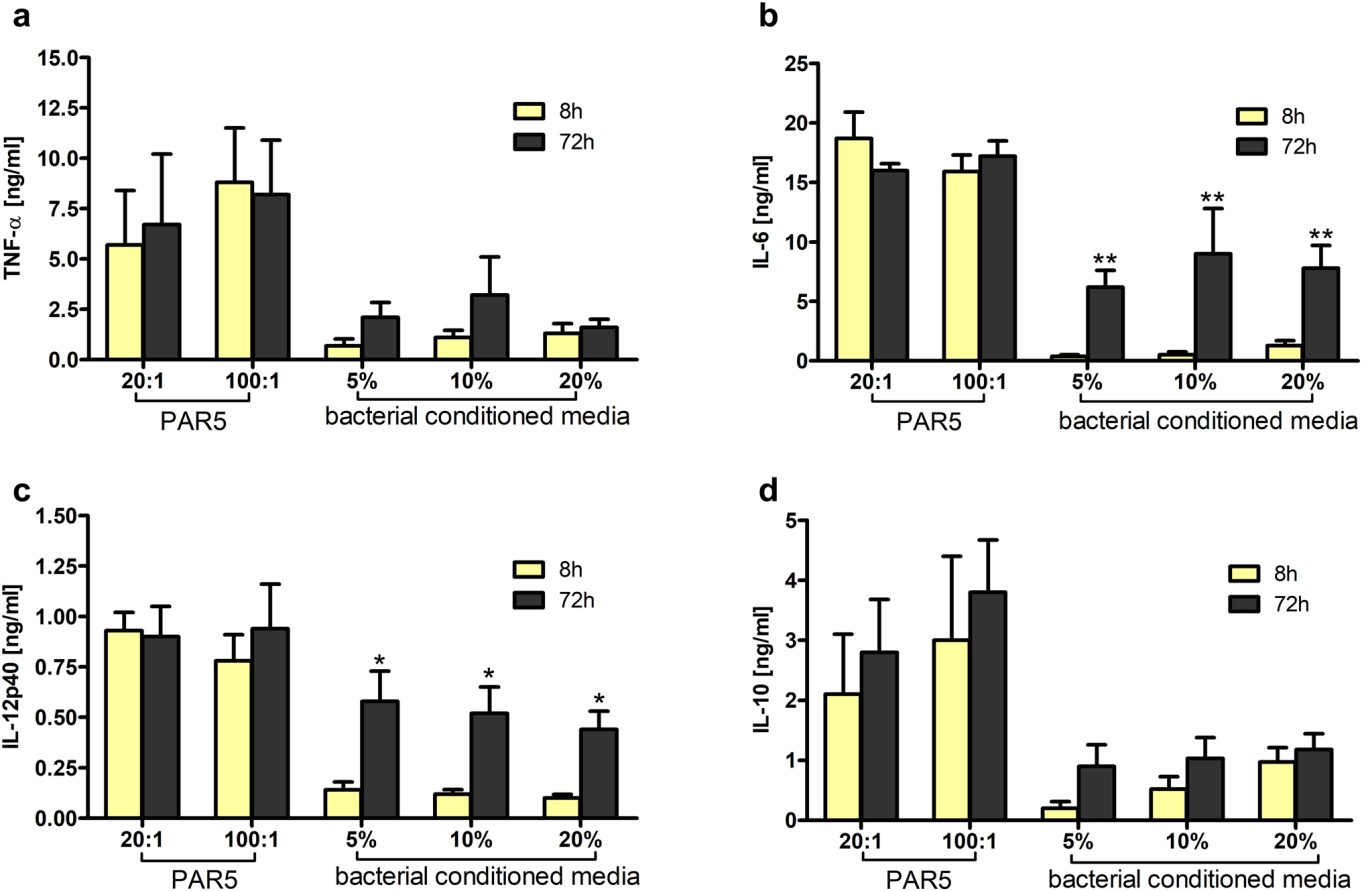
Cytokine production by neutrophils exposed to heat-killed bacteria cells (PAR5) or bacterial-conditioned media (BCM). Levels of TNF-α (**a**), IL-6 (**b**), IL-12p40 (**c**), and IL-10 (**d**) were analyzed by ELISA of supernatants collected from 24-h cultures of neutrophils (5 × 10^5^/well). Neutrophils were activated with tested bacteria at 20:1 and 100:1 ratios of PAR5:neutrophils or with bacterial-conditioned media (5, 10, 20% of total volume). Yellow bars: PAR5-8 h and BCM-8 h. Black bars: PAR5-72 h and BCM-72 h. Cytokine levels in non-stimulated cells were: TNF-α and IL-6 below the detection limit, for IL-10 and IL-12p40, < 0.075 and < 0.035 ng/ml, respectively. Data are mean ± SEM values of three independent experiments. Each group was run in triplicates. *p < 0.05, **p < 0.005 at 8 h vs. 72 h, Student’s *t* test. (Color figure online)

**Fig. 3:**
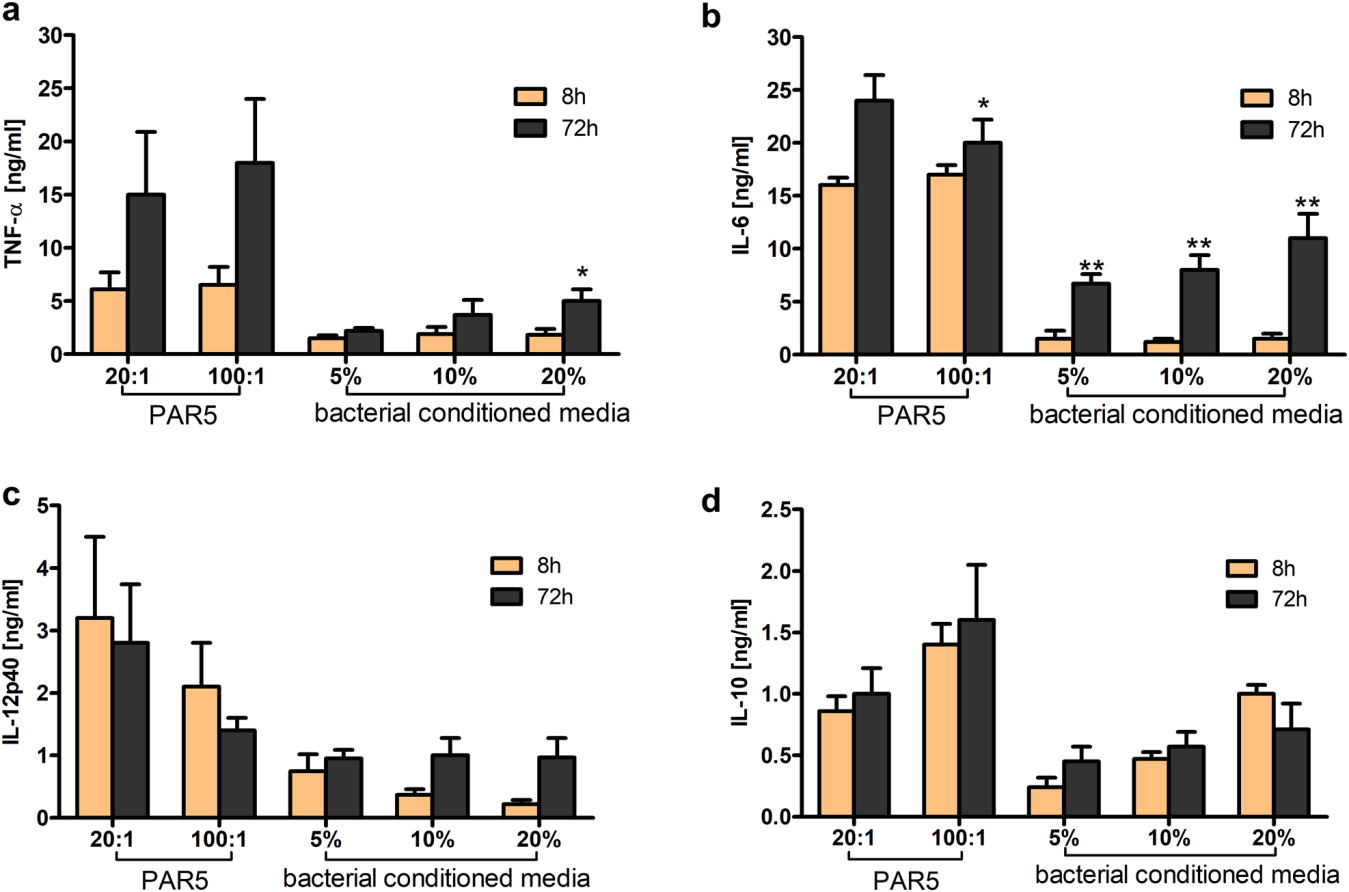
Cytokine production by macrophages exposed to heat-killed bacteria cells (PAR5) and bacterial-conditioned media (BCM). Levels of TNF-α (**a**), IL-6 (**b**), IL-12p40 (**c**), and IL-10 (**d**) were analyzed by ELISA of supernatants collected from 24-h cultures of macrophages (5 × 10^5^/well). Macrophages were activated with tested bacteria used at 20:1 and 100:1 ratios of PAR5:macrophages or with bacterial-conditioned media (5, 10, 20% of total volume). Orange bars: PAR5-8 h and BCM-8 h. Black bars: PAR5-72 h and BCM-72 h. Cytokine levels in non-stimulated cells were: TNF-α < 0.036 ng/ml, IL-6 below the detection limit, for IL-10 < 0.010 ng/ml and IL-12p40, < 0.180 ng/ml. Data are mean ± SEM values of three independent experiments. Each group was run in triplicates. *p < 0.05, **p < 0.005 for 8 h vs. 72 h, Student’s *t* test. (Color figure online)

As shown in Fig. 2, neutrophils stimulated with PAR5-72 h bacterial cells produced similar amounts of all tested cytokines (TNF-α, IL-6, IL-12p40, and IL-10) as those stimulated with PAR5-8 h bacteria. Macrophages, cultured under the same conditions, showed a trend towards higher production of TNF-α and IL-10, and a significantly higher production of IL-6 when stimulated with biofilm PAR5-72 h bacterial cells (Fig. 3). The ratio of bacteria to activated target cells (MOI—multiplicity of infection of 100:1 vs. 20:1 bacteria per cell) had no substantial impact on the cytokine profile or total production.

On the other hand, comparison of bacterial supernatants, rather than cells, showed a different pattern. Neutrophils stimulated with BCM-72 h released significantly more IL-6 and IL-12p40 than those stimulated with BCM-8 h (Fig. 2). The level of IL-6 in neutrophil cultures exposed to BCM-72 h was more than tenfold higher than the level of IL-6 stimulated with BCM-8 h (Fig. 2b). The stimulation of macrophages with BCM-72 h showed a massive release of IL-6, and a trend towards increased IL-12p40 production (Fig. 3).

Importantly, the IL-6:IL-10 ratio reached 15:1 for macrophages and 7:1 for neutrophils, suggesting a strong proinflammatory state of activation. An enhanced TNF-α:IL-10 ratio in supernatants collected from the cells exposed to BCM-72 h (3:1 for neutrophils, 6:1 for macrophages) confirms the proinflammatory profile of these cells.

**Fig. 4:**
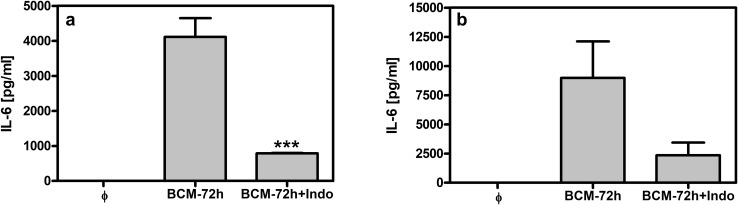
Influence of indomethacin (Indo) on IL-6 secretion by neutrophils and macrophages stimulated with BCM-72 h. Neutrophils (**a**) and macrophages (**b**) at 5 × 10^5^/well were stimulated with BCM-72 h (20%) in the presence or absence of Indo (10 µM). IL-6 level was analyzed in supernatants collected from 24-h cell cultures by ELISA. φ—non-stimulated cells, expressed IL-6 release below 0.1 ng/ml. Data are mean ± SEM values of three independent experiments. Groups were run in triplicates. ***p < 0.001 for BCM-72 h vs. BCM-72 h + Indo, one-way ANOVA

Moreover, the inhibition of prostaglandins production with indomethacin significantly reduced the production of IL-6, suggesting a positive proinflammatory feedback between these two mediators may operate (Fig. 4a, b).

### iNOS, Arg-1, and COX-2 expression in cells exposed to *P. aeruginosa* PAR5

In parallel to determining cytokine secretion, we measured expression levels of iNOS, COX-2 and Arg-1. iNOS and COX-2 are proinflammatory enzymes responsible for productions of NO and PGE_2_, respectively, whereas Arg-1 is a key marker of immunoregulatory N2 neutrophils and M2 macrophages [22, 32].

**Fig. 5:**
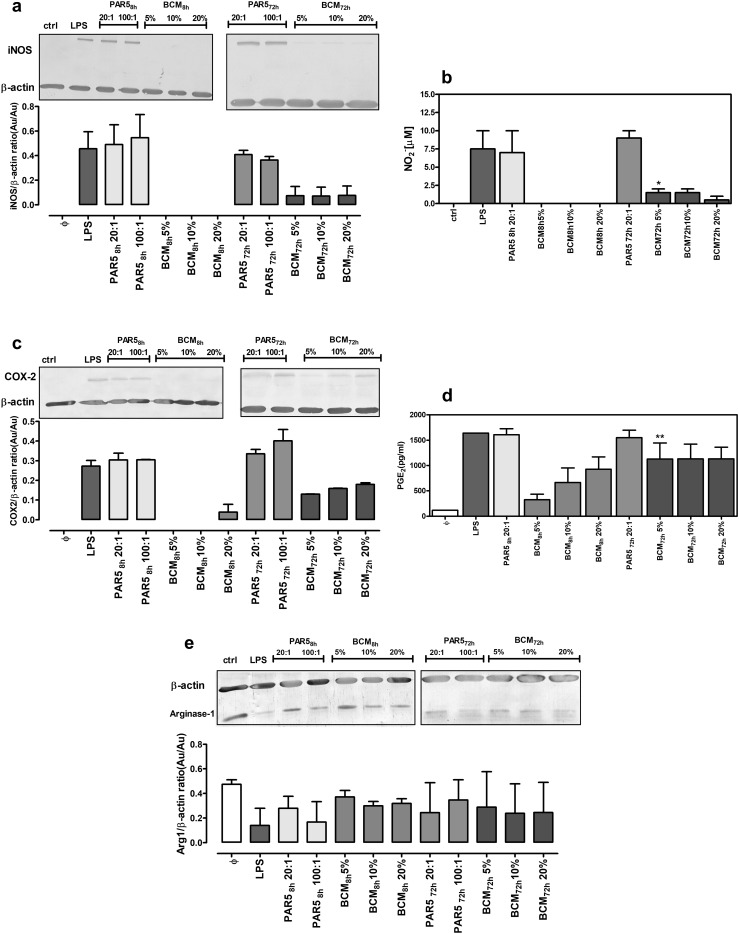
Expression of iNOS, COX-2, and Arg-1 proteins in neutrophils exposed to PAR5 or bacterial-conditioned media. Expression levels of iNOS (**a**), COX-2 (**c**), and Arg-1 (**e**) were measured by western blot analysis of cell lysates collected from 24-h cultures of neutrophils (5 × 10^5^/well). Neutrophils were activated with tested bacteria at a ratio of 20:1 and 100:1 of PAR5:neutrophils, with bacterial-conditioned media (the % of total volume) or with LPS (0.1 µg/ml) as a control. Densitometric analysis of bands from two experiments is shown. Data are normalized to constitutive expression levels of β-actin. In the same experiments, levels of NO_2_ (**b**) and PGE_2_ (**d**) in supernatants were measured. *p < 0.05, **p < 0.005 for 5% BCM-8 h vs. 5% BCN-72 h, one-way ANOVA

**Fig. 6:**
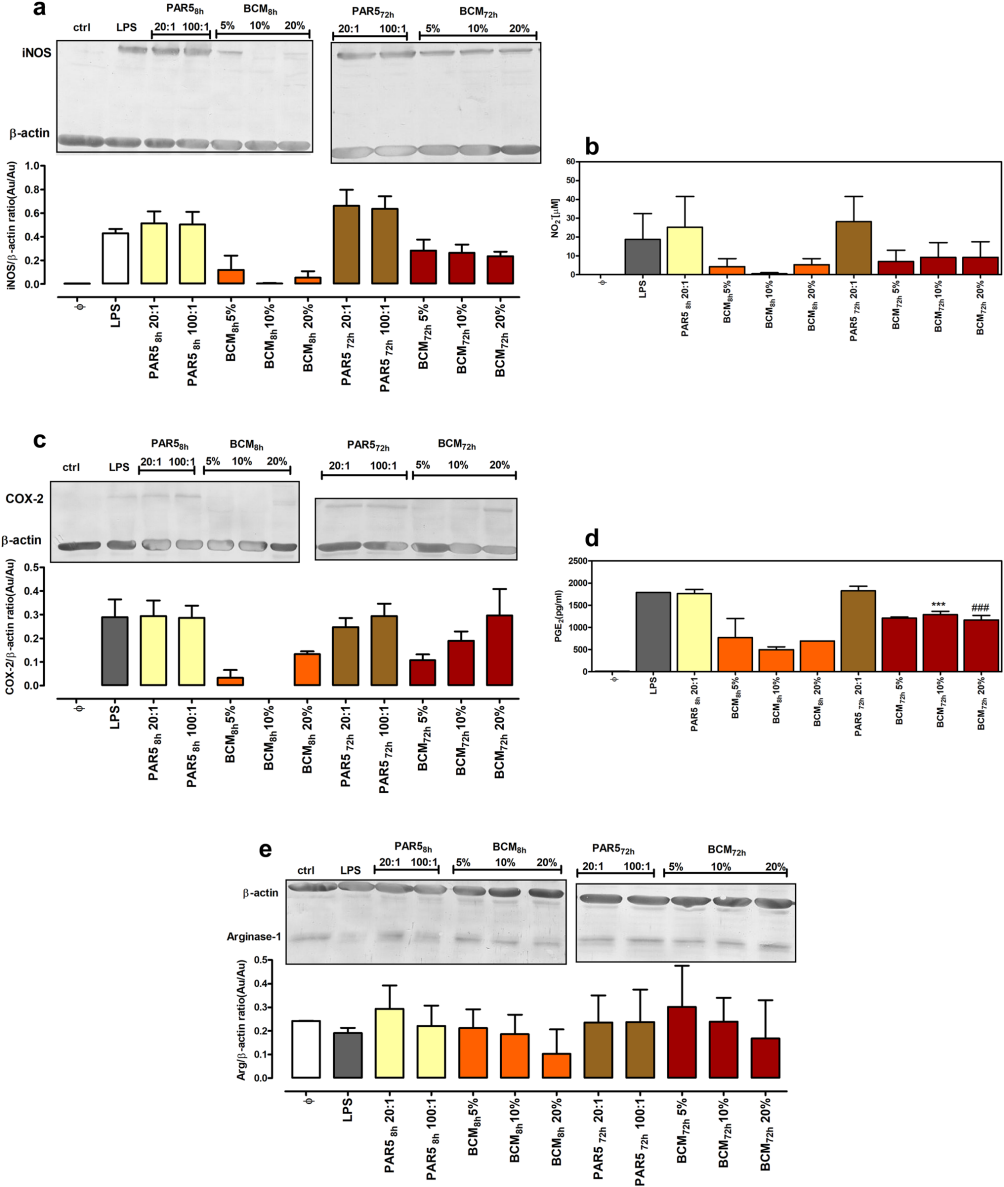
Expression of iNOS, COX-2 and Arg-1 proteins in macrophages exposed to PAR5 or bacterial-conditioned media. Expression levels of iNOS (**a**), COX-2 (**c**), and Arg-1 (**e)** were measured by western blot analysis of cell lysates collected from 24-h cultures of macrophages (5 × 10^5^/well). Macrophages were activated with tested bacteria at a ratio of 20:1 and 100:1 of PAR5:macrophages, with bacterial-conditioned media (the % of total volume) or with LPS (0.1 µg/ml) as a control. Densitometric analysis of bands from two experiments is shown. Data are normalized to constitutively expressed β-actin levels. In the same experiments, levels of NO_2_ (**b**) and PGE_2_ (**d**) in supernatants were measured. ***p < 0.001 for 10% BCM-8 h vs.10% BCM-72 h, ^###^*p* < 0.001 for 20% BCM-8 h vs. 20% BCM-72 h, one-way ANOVA

Stimulation of neutrophils and macrophages with PAR5-8 h and PAR5-72 bacteria caused a massive induction of iNOS and COX-2 proteins in these cells, together with significant secretion of nitrites (stable NO metabolites) and PGE_2_ (> 1.5 ng/ml). There were no differences in Arg-1 expression in cells stimulated with PAR5-8 h and PAR5-72 h (Figs. 5, 6).

Soluble products of *P. aeruginosa* (BCM) produced at early and late phases of culture differentially affected the expression levels of tested enzymes. BCM-8 h did not induce the expression of iNOS or COX-2 in inflammatory cells and the expression of Arg-1 was similar to that of control non-stimulated cells (Figs. 5, 6). In contrast, BCM-72 h induced iNOS and COX-2 expression, similar to stimulation with LPS, the prototypical stimulus for proinflammatory N1/M1 cells. However, neutrophils and macrophages stimulated with BCM-72 h produced substantial amounts of inflammatory PGE_2_ (Figs. 5d, 6d) while the production of microbicidal NO was negligible (Figs. 5b, 6b). All these results point to hyperinflammatory state of activation of the cells exposed to BCM-72 h.

### Effect of LPS and DNA inhibitors on stimulatory properties of BCM-72 h

While BCM-72 h showed a similar capacity to induce the production of proinflammatory mediators as whole bacterial cells of PAR5, BCM-8 h showed only weak stimulatory properties. These observations suggest that BCM-8 h contains low subactive concentrations of active extracellular bacterial biofilm products, consistent with previously reported growth rate of biofilm by *P. aeruginosa* [11, 23, 33, 34]. As shown in Fig. 1b, a substantial amount of EPS produced by PAR5 was observed after 24 h of biofilm formation, followed by a plateau (48–72 h) and a breakdown of biofilm mass after 72 h of culture. In contrast, after 8 h, the EPS amount was negligible and cells could be considered as planktonic cells not hidden in a biofilm matrix. Moreover, BCM-8 h contained several times lower concentrations of LPS and DNA than BCM-72 h (Fig. 1b). This might explain weak stimulatory activity of BCM-8 h.

We, therefore, examined the contribution of different components of BCM-72 h to the activation of phagocytes in more detail. For this purpose, we focused on neutrophils, the major infiltrating cells in severe *P. aeruginosa* infections. First, we excluded the contribution of small molecules, such as QS molecules, in the biological activity of BCM-72 h. Fractions of BCM-72 h with MW below 10 kDa were not able to stimulate phagocytes for the production of cytokines. By contrast, the fractions with MW above 10 kDa showed the same stimulatory capacity as intact BCM-72 h (data not shown). Therefore, bacterial components of high MW, such as extracellular DNA, LPS and EPS, were the major candidates to be held responsible for the proinflammatory properties of BCM-72 h.

**Fig. 7:**
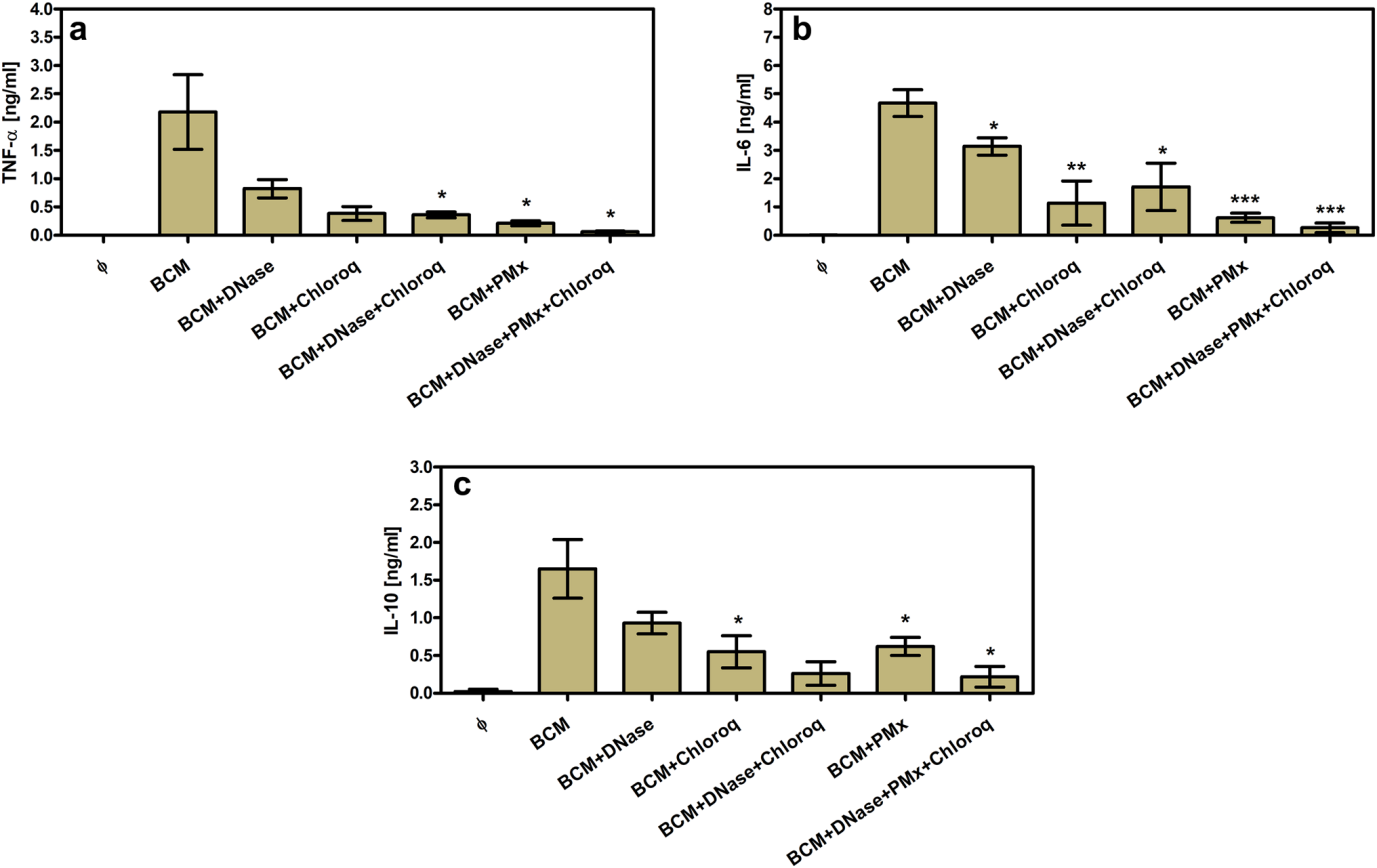
Effect of LPS and DNA inhibitors on stimulatory properties of BCM-72 h. Neutrophils at 5 × 10^5^/well were cultured with 20% BCM-72 h: (i) untreated, (ii) treated with DNA inhibitors (DNase and/or chloroquine), (iii) treated with inhibitor of LPS (PMx), (iv) treated with all tested inhibitors. TNF-α (**a**), IL-6 (**b**) and IL-10 (**c**) levels were analyzed by ELISA in supernatants collected from 24-h cell cultures. Data are mean ± SEM values of three independent experiments. Each group was run in triplicates. *p < 0.05, **p < 0.005, ***p < 0.001 between treated and untreated BCM-72 h, one-way ANOVA

Addition of DNase I and chloroquine (an endosomal acidification blocker and selective inhibitor of the DNA/TLR9 pathway) [35], or polymyxin b (PMx, an inhibitor of LPS [36]) markedly blocked the ability of BCM to stimulate cytokine production by neutrophils (Fig. 7a–c). Importantly, the mixture of LPS and DNA inhibitors decreased both TNF-α and IL-6 production by more than 95%. This indicates that EPS did not contribute to BCM-72 h activity and suggests that EPS present in 20% BCM-72 h was at non-stimulatory concentrations.

### Stimulatory properties of purified LPS, DNA and EPS isolated from BCM-72 h

To support the above observations, neutrophils were exposed to pure LPS, DNA and EPS isolated from BCM-72 h. First, we have estimated the lowest effective concentrations of the agents. All tested agents markedly stimulated IL-6 secretion from neutrophils at concentrations above 100 ng/ml for LPS, 3 µg/ml for DNA and 30 µg/ml for EPS (data not shown).

**Fig. 8:**
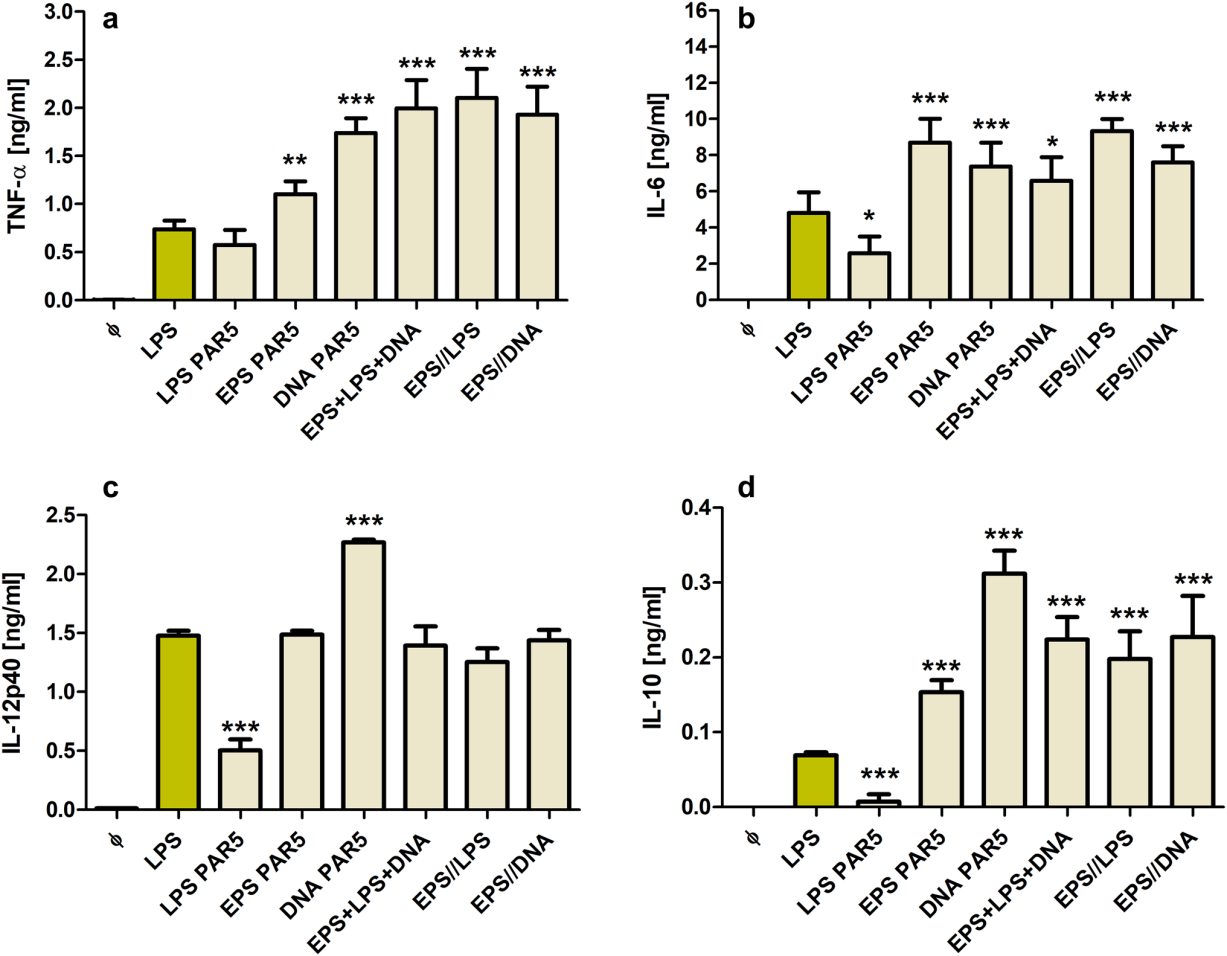
Cytokine production by neutrophils exposed to agents isolated from BCM-72 h. Levels of TNF-α (**a**), IL-6 (**b**), IL-12p40 (**c**), and IL-10 (**d**) were analyzed by ELISA of supernatants collected from 24-h cultures of neutrophils (5 × 10^5^/well). Neutrophils were stimulated with tested agents isolated from PAR5 BCM at concentrations: LPS—100 ng/ml, EPS—30 µg/ml and DNA—3 µg/ml. In two last groups, the cells were preincubated for 1 h with EPS and then re-stimulated with LPS or DNA. After 24 h, supernatants were collected and the amounts of cytokines were estimated. Data are mean ± SEM values of three independent experiments. Each group was run in triplicates. As a control, LPS from *E. coli* was used (LPS, 100 ng/ml). *p < 0.05, **p < 0.005, ***p < 0.001 vs. LPS *E.coli*, one-way ANOVA

Then, the activity of the agents, used at the above concentrations, was compared with the activity of *E. coli* LPS (100 ng/ml), referential stimulator of inflammatory mediators. As shown in Fig. 8, neutrophils exposed to DNA secreted significantly more tested cytokines than those stimulated with *E. coli* LPS. Importantly, the effect was observed at the concentration of DNA similar to that observed in BCM-72 h used in our experimental models (10% BCM-72 h).

By contrast, PAR5 LPS was weaker stimulator of neutrophil cytokine production than *E. coli* LPS. However, it favors the production of proinflammatory cytokines (TNF-α:IL-10 ratio was 50:1 for PAR5 LPS and 10:1 for *E.coli* LPS).

EPS showed stimulatory properties similar to DNA but at the concentration much higher than that estimated in BCM 72 h—20%. Importantly, joint effect of the agents on the production of proinflammatory cytokines (TNF-α, IL-6) was similar to that achieved by stimulation with DNA alone. Moreover, preincubation of neutrophils with EPS, the primarily macromolecule of biofilm matrix, did not alter the neutrophil capacity to secrete inflammatory mediators after LPS and DNA stimulation (Fig. 8).

## Discussion

Biofilm formation in the course of *P. aeruginosa* infections is associated with the development of chronic inflammation and pathogen persistence [5]. In this study, we demonstrate that biofilm is a rich source of concentrated extracellular phagocyte-activating bacterial components, including DNA, LPS and EPS. Importantly, our knowledge of bacteria–neutrophil interactions has evolved since discoveries of the different immunostimulatory properties of planktonic and biofilm forms of pathogens [8, 37]. Bacterial infections start with planktonic growth followed by a formation of biofilm. More importantly, interactions of bacteria with neutrophils at planktonic stage of infections usually result in pathogen killing, controlled production of inflammatory mediators, neutrophil apoptosis and the resolution of acute inflammation. On the contrary, the biofilm stage of bacterial infection favors necrosis of neutrophils and causes chronic inflammation [8, 21].

Chronic *P. aeruginosa* infection is the outcome of a complex series of changes in *P. aeruginosa* phenotype. Infection with “wild-type” strains of *P. aeruginosa* is characterized by the proliferation of planktonic form of bacteria cells. Their long persistence is associated with complex mechanisms of adaptation and shift from planktonic to biofilm phenotypes. Moreover, biofilm-type pathogens undergo genetic, phenotypic and physiological diversification [38]. These biofilm-forming strains colonize infected tissue and exist at various stages of biofilm development in numberless macrocolonies [39]. Such biofilm lifestyle cycle lasts 4–10 days and can be subdivided into following steps: attachment or aggregation of bacterial planktonic cells, formation of biofilm matrix, biofilm maturation, cell detachment and dispersion. Finally, such free-living biofilm cells, misleadingly termed as planktonic cells, spread and colonize host tissues to form biofilms at new sites [40]. Herein, in this study, all experiments were performed using the selected mucoid bacterial strain of *P. aeruginosa* showing a massive production of biofilm components.

The main objective of this study was to show that neutrophils and macrophages can be effectively stimulated by *P. aeruginosa*-derived biofilm extracellular substances without direct contact with bacterial cells, because the biofilm provides a very concentrated stable source of PAMPS which are otherwise present at very low concentrations in bacterial culture supernatant. We further hypothesize that the presence of such stimulated cells (BANs, BAMs) in a hyperinflammatory state of activation may explain the detrimental role of neutrophils in chronic *P. aeruginosa* infection associated with the pathogenesis of CF [41, 42] and, as we have previously observed, in chronic rhinosinusitis [15].

We explored the role of the biofilm using, as a model, conditioned medium from a high biofilm producer strain of *P. aeruginosa*, and as responding innate immune cells murine macrophages and neutrophils. As we expected, 72 h, but not 8-h cultures of *P aeruginosa* PAR5 were a rich source of biofilm components, including DNA, LPS and EPS. Importantly, the density of PAR5 in our experimental model of bacterial cultures (10^7^—5 × 10^9^ CFU/ml) was similar to *P. aeruginosa* sputum densities demonstrated in cystic fibrosis patients [43]. The density of infiltrated neutrophils in CF sputum [44] is 10–20 times higher than the density of in vitro-stimulated leukocytes (5 × 10^5^/ml). Therefore, one may expect even stronger hyperinflammatory response in vivo.

Conditioned medium from early cultures of PAR5, which contained small amounts of biofilm products, showed a weak stimulation of either neutrophils or macrophages. The lack of biofilm components at this time point is in agreement with the previously observed growth rate of *P. aeruginosa* biofilms and scant release of EPSs at this stage of biofilm development [23, 45].

However, when the PAR5 culture was extended to 72 h, biofilm production could easily be detected. In parallel, the conditioned medium (BCM-72 h) acquired strong stimulatory properties towards both neutrophils and macrophages. Specifically, the conditioned medium induced the release of TNF-α, IL-6, IL-12p40, NO, and PGE_2_. Extremely high release of IL-6 was observed from targeted cell types, resulting in a high ratio of IL-6/IL-10, well-known pro- and anti-inflammatory cytokines. To confirm a causal link between the increased concentrations of DNA and LPS associated with biofilm formation, and the immunostimulatory properties of the conditioned medium we removed DNA using DNAse enzyme, and blocked LPS activity using polymyxin B. Both these components strongly contributed to the proinflammatory activity of the conditioned medium, as their inhibition almost completely abolished the activity of BCM-72 h. On the other hand, the incubation of neutrophils with pure DNA and LPS isolated from BCM-72 h showed that both agents, at concentrations close to those at 20% BCM-72 h, markedly stimulated proinflammatory mediators.

Our results are in agreement with previous studies reported that components of biofilm matrix exert proinflammatory properties associated with the pathogenicity of biofilm-forming *P. aeruginosa*. These components primarily include QS molecules [9, 46], EPSs [10, 34], and finally, LPS and extracellular bacterial DNA, two major virulence and proinflammatory factors of *P. aeruginosa* biofilms [13, 33, 47, 48]. We excluded the effect of QS molecules from our experiments because there was no BCM-72 h activity in the low MW fraction (< 10 kD). In addition, a combined neutralization of DNA and LPS completely blocked BCM-72 h activity. Such blockade excluded the significant contribution of other agents including EPS. Why EPS, the major component of *P. aeruginosa* biofilm, was not active? One possibility is that EPSs have a non-effective concentration in BCM-72 h. Indeed, we have shown that EPSs isolated from PAR5 BCM-72 h stimulate cytokine production by neutrophils at high concentrations (> 30 µg/ml) which were not achieved in the tested bacterial-conditioned media (< 20% BCM-72 h). Nevertheless, *P. aeruginosa-*derived EPS (e.g., alginate) may achieve very high concentrations in vivo at chronic inflammation sites [49]. Therefore, in vivo, EPS not only protects bacteria hidden in a biofilm matrix against the immune attack, but also traps extracellular LPS and DNA to maintain their long-term effective immunostimulatory concentrations. Importantly, DNA and LPS, in contrast to EPS, can effectively activate inflammatory cells at very low concentrations (< 1 µg/ml) [48]. Indeed, in our experimental conditions, DNA and LPS, the major components of BCM-72 h, independently stimulated the massive cytokine production in neutrophils. These findings are consistent with previous observations that extracellular DNA, apart from its role in biofilm composition, is an extremely active component of *P. aeruginosa* biofilms. Extracellular DNA triggers neutrophil activation through TLR9-dependent/-independent mechanisms, causes target cell death by necrosis and exacerbates chronic *P. aeruginosa* infections [12, 35, 48]. Moreover, it has been reported that DNA can initiate lung injury by stimulating neutrophil–endothelial cell interactions predisposing to the increase in endothelial permeability [35].

Interestingly, the massive production of IL-6 and PGE_2_ is in response to PAR5 stimulation. This observation confirms a role of the PGE_2_/IL-6 axis in *P. aeruginosa* pathogenicity. Indeed, IL-6 levels have been reported to be increased markedly in cystic fibrosis patients when the disease exacerbates [50–52]. Moreover, it has been demonstrated that IL-6 enhances mouse mortality in *P. aeruginosa* pulmonary infections [35, 52, 53]. The PGE_2_/IL-6 axis promotes IL-23 (IL-12p40), inhibits IL-12p70, and subsequently induces production of IL-17, the cytokine responsible for exacerbation of chronic inflammatory processes [52, 54, 55]. PGE_2_ also contributes to the suppression of innate immunity during chronic mucosal bacterial infections [56–58].

**Fig. 9:**
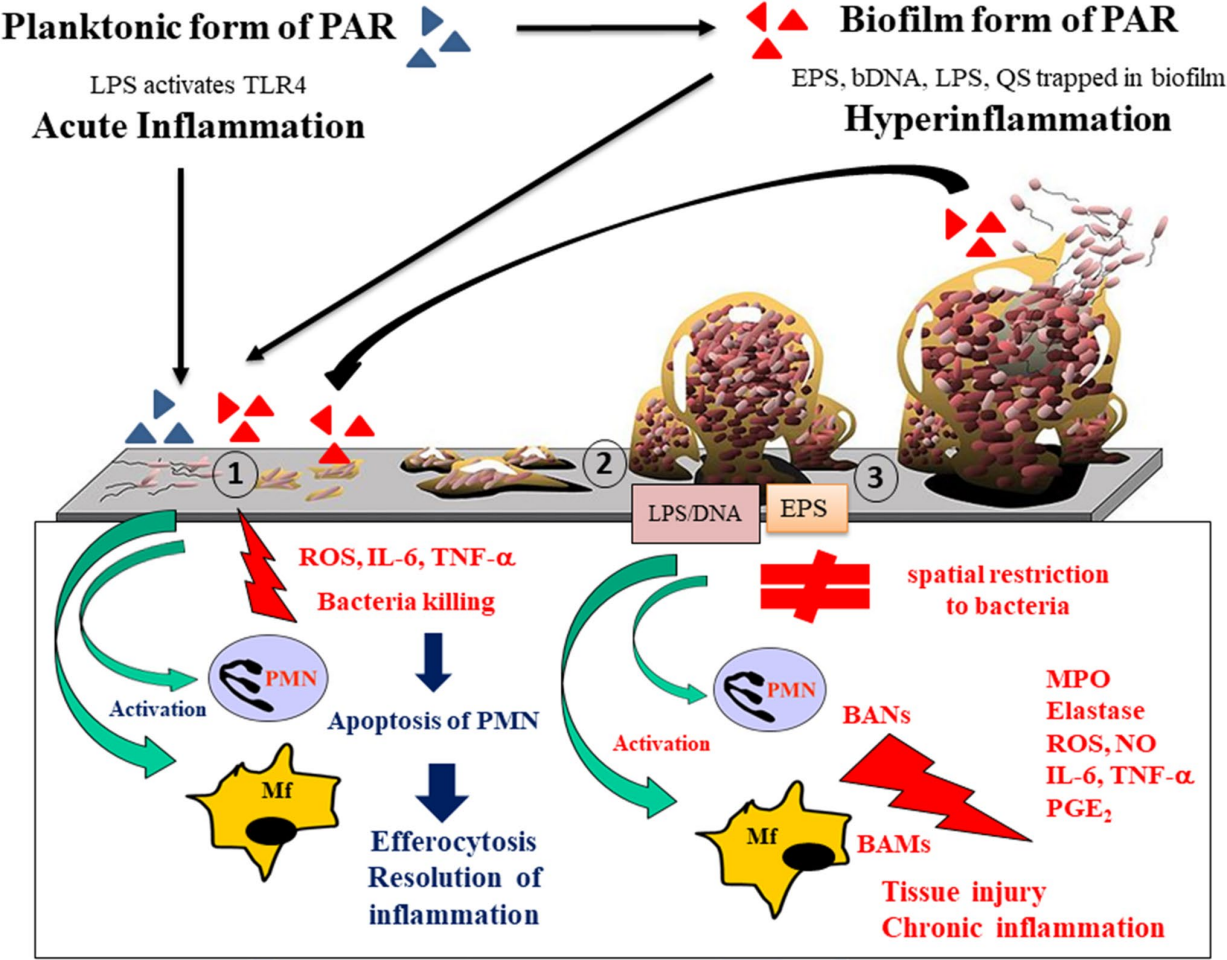
Proposed model for cross talk between *P. aeruginosa* (PAR) and innate immunity (neutrophils and macrophages) at a site of acute and chronic inflammation. Acute infection and acute inflammation: at an early stage of infection planktonic form of *P. aeruginosa* induces an inflammatory response through TLRs at the site of bacteria entry. Activation of infiltrating neutrophils results in the production of inflammatory mediators and stimulation of phagocytosis, which kills planktonic bacterial cells. After eliminating pathogens, neutrophils die by apoptosis and are silently phagocytosed (neutrophil efferocytosis) by macrophages. Finally, acute infection and inflammation are terminated. However, if pathogens are not killed they will attach to host cells, build a biofilm and chronic infection will develop. Chronic infection and chronic inflammation: bacteria that acquired biofilm-type properties can survive as sessile cells in a few days long biofilm cycle (biofilm development stages: (1) irreversible attachment of bacteria to infected host cells or aggregation in body fluids, (2) biofilm formation and maturation, (3) bacteria cell dispersion, colonization of other sites, building one to three new biofilms). Formation of a biofilm matrix is accompanied by the local accumulation of EPSs, DNA, and LPS molecules reaching immunostimulatory concentrations. In the course of biofilm maturation, extracellular LPS and DNA seem to be major stimulators of neutrophils (BANs) and macrophages (BAMs), while EPS acts as a physical barrier and as a trap for other biofilm matrix components. The interaction between biofilm and BANs/BAMs might, therefore, favor hyperinflammation associated with tissue injury rather than effective killing of pathogens and resolution of acute inflammation. Such scenario is in agreement with clinical reports showing massive neutrophil infiltration in chronic *P. aeruginosa* infections with concomitant release of detrimental substances such as elastase, ROS, NO, prostaglandins and proinflammatory cytokines

Taken together, our finding partially explain why neutrophils infiltrating chronically infected tissue (e.g., CF airways) are not able to clear *P. aeruginosa* biofilm and contribute to tissue damage by releasing their dangerous cargo, as demonstrated in clinical studies [59]. This in vitro study showed that biofilm matrix components (LPS, DNA, EPS), without direct contact of bacteria and with phagocytes, stimulated release of proinflammatory mediators. Therefore, the interaction between biofilm and BAN/BAMs might favor chronic inflammation rather than effective killing of pathogens (“frustrated phagocytosis”) and resolution of inflammation (Fig. 9).

## Data Availability

The data that support the findings of this study are available from the corresponding author upon reasonable request.
